# Poor Access for African Researchers to African Emergency Care Publications: A Cross-sectional Study

**DOI:** 10.5811/westjem.2017.8.34930

**Published:** 2017-09-11

**Authors:** Stevan R. Bruijns, Mmapeladi Maesela, Suniti Sinha, Megan Banner

**Affiliations:** * University of Cape Town, Division of Emergency Medicine, Cape Town, South Africa; † University of Cape Town, Faculty of Health Sciences, Cape Town, South Africa; ‡ African Federation for Emergency Medicine, Cape Town, South Africa

## Abstract

**Introduction:**

Based on relative population size and burden of disease, emergency care publication outputs from low- and middle-income regions are disproportionately lower than those of high-income regions. Ironically, outputs from regions with higher publication rates are often less relevant in the African context. As a result, the dissemination of and access to local research is essential to local researchers, but the cost of this access (actual and cost-wise) remains unknown. The aim of this study was to describe access to African emergency care publications in terms of publisher-based access (open access or subscription) and alternate access (self-archived or author provided), as well as the cost of access.

**Methods:**

We conducted a retrospective, cross-sectional study using all emergency medicine publications included in Scopus between 2011 and 2015. A sequential search strategy described access to each article, and we calculated mean article charges against the purchasing power parity index (used to describe out-of-pocket expense).

**Results:**

We included 666 publications from 49 journals, of which 395 (59.3%) were open access. For subscription-based articles, 106 (39.1%) were self-archived, 60 (22.1%) were author-provided, and 105 (38.8%) were inaccessible. Mean article access cost was $36.44, and mean processing charge was $2,319.34. Using the purchasing power parity index it was calculated that equivalent out-of-pocket expenditure for South African, Ghanaian and Tanzanian authors would respectively be $15.77, $10.44 and $13.04 for access, and $1,004.02, $664.36 and $830.27 for processing. Based on this, the corrected cost of a single-unit article access or process charge for South African, Ghanaian and Tanzanian authors, respectively, was 2.3, 3.5 and 2.8 times higher than the standard rate.

**Conclusion:**

One in six African emergency care publications are inaccessible outside institutional library subscriptions; additionally, the cost of access to publications in low- and middle-income countries appears prohibitive. Publishers should strongly consider revising pricing for more equitable access for researchers from low- and middle-income countries.

## INTRODUCTION

Given the stark differences in emergency care resource requirements and availability between high-income countries and low- and middle-income countries, it is important that African emergency care research is both conducted and disseminated in accessible format within Africa. Evidence-based care from emergency care research conducted elsewhere often does not apply directly in the African context as the former assumes access to resources that does not translate to the latter.^1^ Publications from the African region made up only 1.8% (829 of 46,901) of global emergency medicine (EM) publication output between 2010 and 2015; but this number is not representative of the relative size of the African continent, nor its relative higher burden of disease, morbidity and mortality compared to other world regions.^2^,^3^ This can be attributed, at least in part, to the small, local, emergency care academic community and the apparent lower interest of emergency care journals (mainly representing high-income settings) in research from low- and middle-income settings.^2^

The fact that around three-quarters of African emergency care publications are published in international journals suggests a necessarily high rate of collaboration – indeed 40% of African emergency care publications included authors from outside Africa, while only 12% of collaboration came from other African countries.^4^–^7^ Thus taking into account a local, developing, emergency care knowledge economy and the relative irrelevance of high-income setting-produced research, the importance of sharing local African-related emergency care research within Africa should be a given. It is, however, not known how much of the continent’s scientific emergency care output is readily accessible to the local emergency care community. The aim of this study was to describe access to African emergency care publications (published between 2011 and 2015) in terms of publisher-based access (open access or subscription) and alternate access (self-archived or author provided), as well as the cost of access or publication (article access, or processing charges, respectively).

**Population Health Research Capsule**

What do we already know about this issue?

Open access publication was founded on the principle that access to research should be universal. Despite this, universal access is not yet a given everywhere.

What was the research question?

What access (cost or otherwise) do African researchers have to African emergency care-related publications?

What was the major finding of the study?

One in six African emergency care publications are inaccessible to African

researchers due to firewalls and cost.

How does this improve population health?

Accessible research is a key contributor to a stable knowledge economy – better access means better knowledge translation, which impacts population health outcomes.

## METHODS

**Figure 1:**
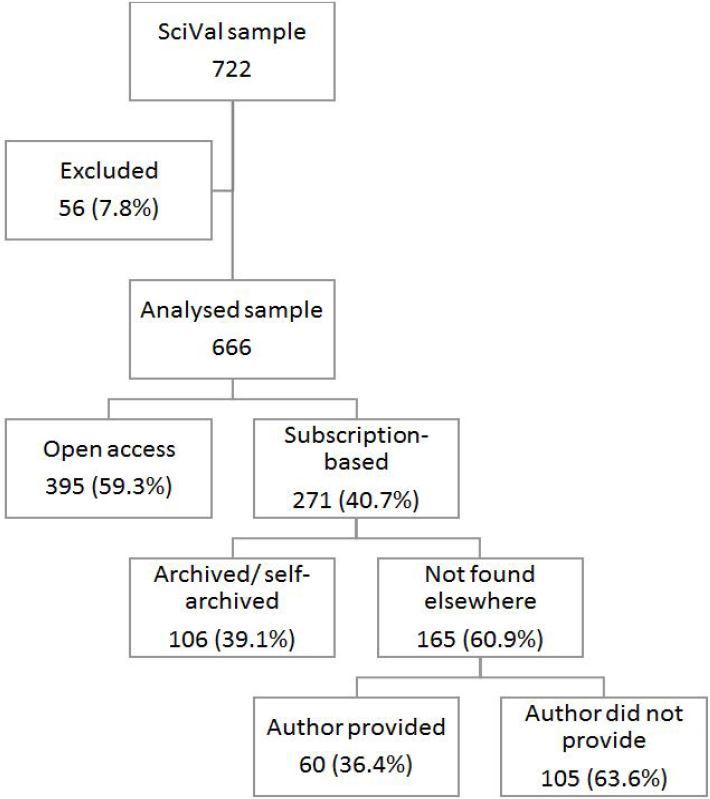
Sample derivation and main findings of the study (numbers of published articles).

We employed a cross-sectional study design, using retrospective, secondary published data from the Scopus database (Elsevier, Amsterdam), supplemented by prospective data solicited from corresponding authors of a smaller cohort of these publications (Figure 1). We included all 722 African EM publications included in the Scopus database between 2011 and 2015, and performed the initial analysis using SciVal (Elsevier, Amsterdam). SciVal is a subscription-based data manager that interacts with Scopus, allowing detailed analysis through automated keyword searches around various aspects of publications from the five years available at the time of data extraction. Using SciVal, we extracted publications from the Scopus database from the African region (based on lead author affiliation) and EM specialty (based on keywords provided by the author as well as the journal during the indexing process). The final sample was then further filtered to include only original articles, reviews, meta-analyses, case reports and editorials. We also collected the publication title, journal title, full author list (including affiliations), and the corresponding author name and contact email.

To determine access to the sampled publications we used the following sequential search:

1. We accessed all the respective journal websites, noting whether publications were either open access or subscription based. Article process charges (for open-access publications) and article access charges (for subscription-based publications) were also collected. Variations in cost, where different charges applied depending on different income regions, were collected as such.
2. We undertook a keyword search (using publication title and author list) using Google, PubMed Central and the following commercial repositories: ResearchGate, Mendeley, and Academia.edu for self-archived copies of either publisher or post-print versions of the subscription-based publications. The publisher version of a publication is the version that is published on the official journal website following copy-editing. The post-print version of a publication is the final manuscript that was accepted for publication following peer-review, but prior to copy-editing. We did not include a search of the torrent site Sci-Hub, given that it would automatically obtain any requested publication not yet in its repository through a non-transparent, widely-considered controversial process (see “Discussion”).^8^
3. Finally, for any subscription-based, un-archived publications, we sent an email request to respective corresponding authors to share an authorized version of their publication. Articles were then checked and categorized as communicable, non-communicable, injury- or policy-themed publications to provide perspective on publication themes. A hypothetical corrected charge, using the World Health Organization (WHO) purchasing power parity (PPP) index was calculated on article process and access charges.^9^ PPP is based on the hypothesis that similar items should cost the same, irrespective of currency differences, no matter where it is bought in the world. The WHO PPP index describes the deviation from parity using the United States dollar ($) as a baseline. The correction represents the hypothetical out-of-pocket expense for the same goods. We described purchasing power for the following three African countries: South Africa, Ghana and Tanzania. The reasoning for selecting these specific countries was that each offers specialist training in EM, representing a regional, emergency care, research collaboration hub, as well as representing a different income group (high-middle, low-middle and low income, respectively).^10^,^11^ The study received ethical approval through the University of Cape Town’s Human Research Ethics Committee.

We analyzed data using Microsoft Excel (Microsoft Office, Redmond, U.S.) and they are presented as proportions and tables. Article access and process charges are provided in U.S. dollars ($) and described by the mean and standard deviation. We used XE for currency conversions.^12^ Calculations were made using the exchange rates published on December 12, 2016: $1 was respectively worth 13.77 South African rand, 4.30 Ghanaian cedi and 2 176.52 Tanzanian shilling. The PPP index for 2016 was 5.96 for South Africa, 1.23 for Ghana and 779.21 for Tanzania. We used the X^2^-test to test associations between categorical variables (article categories, etc.) with a *p* value less than 0.05 accepted as significance. The 95% confidence interval (CI) was provided to describe precision between continuous variables (article process and access charges, etc.).

## RESULTS

Figure 1 describes how the sample was derived, as well as the main findings of the study. Exclusions consisted of duplications and mis-indexed publications. The final sample consisted of 666 publications from 49 journals. We failed to access 15.8% (105/666) of publications – one of every six publications. The difference in access between open-access and subscription-based publications was significant (X^2^=12.46, p<0.01). The difference in access between self-archived publications and those not found was also significant (X^2^=8.62, p=0.03). But the difference in access between publications provided by the corresponding author and those where the author did not respond was not significant (X^2^=5.24, p=0.16).

**Figure 2:**
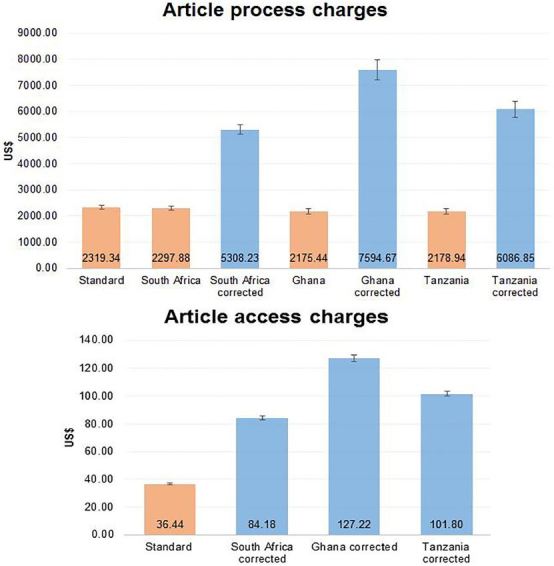
Article-process charges at the top with access charges below. The red bars indicate standard charges and blue bars indicate purchasing power parity-corrected charges. The error bars indicate the 95% confidence interval.

Not all journals charged for article processing or access. The mean article access and process charges by journals that charged for these were $36.44 (standard deviation of $6.05) from 36 journals, and $2,319.34 (standard deviation of $869.90) from 41 journals. The corrected cost of a single-unit article access or process was 2.3, 3.5 and 2.8 times higher than the standard rate, respectively, for South African, Ghanaian and Tanzanian authors. This is graphically presented in Figure 2. The error bars represent the 95% CI and indicate the significant difference between standard charges and PPP-corrected charges.

Discounts and waivers were applied by some journals with regard to article process charges. Thirteen (26.5%) journals exclusively published open access, with a further 29 (59.2%) offering the option to publish open-access options in a subscription-based journal. Of the 13 fully open-access journals, four charged no article processing fee. Articles published via these four journals made up 18.2% (121 articles) of the entire sample, of which one, the *African Journal of Emergency Medicine*, published 117 (17.6%) articles – both the single largest contributor to the sample and the cheapest, given that it also charged no article process fees. Authors from South Africa (high-middle income) were eligible for a full article process-charge waiver from one journal and a reduced charge from another one. Authors from Ghana (low-middle income) were eligible for a full article process charge waiver from 15/42 (35.7%) journals and a reduced charge from another two. Authors from Tanzania (low income) were eligible for a full article process-charge waiver from 16/42 (38.1%) journals and a reduced charge from another one. The Table provides the charges for South African, Ghanaian and Tanzanian authors that would result in similar out-of-pocket spending to the standard rate. Of the open-access journals (including journals with an open- access option), the following provided article processing fees less than the charges described in the table: *Annals of Burns and Fire Disasters* ($104.57), *Western Journal of Emergency Medicine* ($400, full waiver for Ghana and Tanzania) and the *Journal of Emergencies, Trauma and Shock* ($600). Of the subscription-based journals, only *Traumatology Journal*, an acute psychiatry journal, provided article access less than the charges described in the table ($11.95).

African and non-African first authors were respectively responsible for 70.3% (468) and 29.7% (198) of all publications. The majority of publications were either injury-themed, 40.7% (271), or policy-themed, 39.9% (266). Supplement A provides detail of the publication themes. African first authors were more likely to publish open access although the difference was not significant (X^2^=3.24, p=0.07). Non-African first authors’ publications were more likely to be found elsewhere (repositories, etc.) but again the difference was not significant (X^2^=2.03, p=0.15). Non-African first authors were also more likely to provide their publications on request but the difference was not significant (X^2^=0.41, p=0.52). Supplement B provides detail of access to publications comparing African and non-African first authors.

## DISCUSSION

Of the small number of African emergency care articles published between 2010 and 2015, around one in six publications were cost-prohibitive. The cost of accessing subscription-based publications is likely to be prohibitive, even for South Africa, a higher-middle income country. It is telling that only one journal out of 36 provided access charges within the out-of-pocket scope of any of the three African countries. For African emergency care to grow, increased research output is essential to fuel its knowledge economy. Only a small number of higher learning institutions on the continent currently offer training in EM, of which only two offer dedicated research degrees.^11^ Furthermore, local emergency care researchers tend to be clinician-researchers, largely unaffiliated with academic institutions. African academic libraries for their part similarly suffer from having to purchase subscriptions using devalued currencies that result in significantly higher costs when compared to high-income countries’ costs.^13^

Van Hoving et al. reported that 58% of African emergency care researchers struggled frequently with publication access, suggesting that local emergency care researchers are unable to make do given poor access and the significantly higher out-of-pocket expense related to publication.^14^ The combination of these elements creates a perfect storm that threatens the growth and development of the specialty locally. Fortunately, article-processing fee waivers and discounts exist for low-middle and low income countries, which is likely why African authors were able to publish via open access more regularly. It was disappointing to see that authors from South Africa (a higher-middle-income country) would incur a significantly higher out-of-pocket publication cost; higher-middle-income countries are as a rule excluded from waivers and discounts.

Open-access publications (largely contributed to by African first authors) made up the bulk of publications, while self-archiving and author-provided publications were fewer. It is interesting to note that policy-themed publications made up nearly half of all non-African first author publications, suggesting that non-African first authors’ main research contribution appears to be deriving emergency care-related policy – something one would have expected to be more within local authors’ remit. The study did not specifically consider whether non-African first authors collaborated on policy publications with local authors. It is, however, not unusual for publications regarding African matters (including policy) to shun African authors. Ironically, a recent World Bank/ Elsevier collaboration regarding African publication prowess in science, technology, engineering and mathematics did not include any African authors.^15^

There were very few communicable and non-communicable disease-themed publications compared with injury-themed publications (which led the publication tally alongside policy). Injury appears to be a particular research focus in Africa: the WHO’s Decade of Action for Road Safety started in 2011 and this will have had a tangible impact in the region on both policy and publication priority.^16^ The journal *Burns* and *Annals of Burns and Fire Disasters* included the second and fourth most African emergency care publications between 2010 and 2015, respectively.^17^ Yet given the unmistakable rise in non-communicable disease in Africa (along with its increasing contribution to mortality), a deeper focus on emergency care-related research in these fields should be encouraged to avoid being left unprepared.^18^,^19^

An article about access to scientific work for authors from low- and middle-income countries would not be complete without a brief discussion about article access using the torrent site, Sci-Hub. As mentioned in the “Methods” section, we did not include Sci-Hub in our search criteria. It is likely that we would have been able to access the majority of subscription-only articles there. However, it remains a highly controversial route of article access.^20^ In terms of its reach, a recent report in *Science* revealed that Sci-Hub was widely used in low- and middle-income regions, such as Eastern Europe, the Middle East, the Indian subcontinent and Southeast Asia, but not so much in sub-Saharan Africa.^21^ Interestingly, the seemingly low penetration figures provided for sub-Saharan Africa should not necessarily be misconstrued as poor uptake of Sci-Hub in this region.^22^ The ratio of active researchers to population size is substantially lower in this region than anywhere else. If this regional ratio was used as a denominator, it would likely provide a very different view of Sci-Hub usage.

## LIMITATIONS

There were a number of limitations of this study. As we used a convenience sample, the sample was small and unpowered. It is also possible that published research was shared with local emergency care researchers that was not traceable through an Internet-search strategy. The WHO PPP index is based on household expenditure, which in turn is affected by relative market prices, exchange rates, wages, interest rates, etc., of which purchasing article access or processing is but one small element. However, we felt that using an index that took into account a person’s basic needs (as represented by household expenditure) would provide relevant context. It is true that purchasing power is constantly in flux, although for most African states it hasn’t changed much compared to the dollar over the last five years.^9^

This study does not account for access initiatives such as Hinari, a WHO initiative set up along with a number of major publishers to enable journal access to low- and lower-middle income countries. Hinari and the majority of the other initiatives, however, require institutional access, excludes higher-middle income countries (which our study has shown to also have financial limitations) and does not assist with article-process charges. Furthermore, Hinari’s own research shows that the service is not accessible in the very countries it aims to support.^23^ An impact survey published by Hinari in 2014 revealed that despite 902 (88%) respondents agreeing that access to scientific literature was important, and 883 (81%) being aware of the access provided through Hinari, only 492 (48%) had access to it.^23^ Today, however, publishers can easily make use of geo-blocking (Internet-content access control based on geographical location) to provide a more efficient yet still selective access to low- and middle-income countries without the assistance and limited resources of the WHO.

Finally, our search for accessible versions of articles could have been improved if the open access search engines, Unpaywall and Open Access Button, had been used.^24^,^25^ These are browser plug-ins that can identify searched articles and then check whether it is accessible anywhere else. We were not aware of these search engines at the time of data collection. Future studies should include these search engines to optimize searches.

## CONCLUSION

In conclusion, nearly half of African emergency care publications would be inaccessible without local university-library access. As a result, researchers without library access must be content with searching for self-archived publications online or contacting authors for a copy, as the cost of accessing these publications through article-access charges is prohibitive. Given the renewed focus on improving emergency care in low- and middle-income settings this may prove to be highly significant. Currently, low-and middle-income populations, including those in Africa, make up around 85% of the world’s population, of which the vast majority will have similar access problems.^26^ It is also unlikely that access is only restricted in this way to emergency care publications. Publishers should therefore strongly consider revising pricing policies to allow more equitable access to publications for researchers in these regions. Strong advocacy is needed from organizations such as the WHO to ensure that operational agendas correlate with access to information.

## Supplementary Information





## Figures and Tables

**Table:** Representative article access and processing fees for South Africa, Ghana and Tanzania that would result in similar out-of-pocket spending to the United States.

Charge	Standard charge	South Africa	Ghana	Tanzania
Article processing	$2,319.34	$1,004.02	$664.36	$830.27
Article access	$36.44	$15.77	$10.44	$13.04
