# Supplementary material for: Poor Access for African Researchers to African Emergency Care Publications: A Cross-sectional Study
Source: West J Emerg Med. 2017 Sep 11;18(6):1018–24. doi: 10.5811/westjem.2017.8.34930 (PMC5654869; doi:10.5811/westjem.2017.8.34930)
Supplement: Supplementary file 1 [file wjem-18-1018-s001.docx]

Supplement A.

| **Supplement A.** Breakdown of all publications as either communicable disease, non-communicable disease, injury or policy publications | | | | | |
| --- | --- | --- | --- | --- | --- |
| **Variable** | **All publications** | **Communicable disease** | **Non-communicable disease** | **Injury** | **Policy** |
| All publications | 666 | 41 (6.2%) | 88 (13.2%) | 271 (40.7%) | 266 (39.9%) |
| Open access | 395 | 27 (6.8%) | 59 (14.9%) | 139 (35.2%) | 170 (43.0%) |
| Subscription-based | 271 | 14 (5.2%) | 29 (10.7%) | 132 (48.7%) | 96 (35.4%) |
| Archived/ self-archived | 106 | 3 (2.8%) | 9 (8.5%) | 63 (59.4%) | 31 (29.2%) |
| Not archived | 165 | 11 (6.7%) | 20 (12.1%) | 69 (41.8%) | 65 (39.1%) |
| Author provided | 60 | 3 (5.0%) | 3 (5.0%) | 28 (46.7%) | 26 (43.3%) |
| Author did not provide | 105 | 8 (7.6%) | 17 (16.2%) | 41 (39.0%) | 39 (37.1%) |
| All publications found | 561 | 33 (5.9%) | 71 (12.7%) | 230 (41.0%) | 227 (40.4%) |
| African first author | 468 | 27 (5.8%) | 71 (15.2%) | 198 (42.3%) | 172 (36.8%) |
| Non-African first author | 198 | 14 (7.1%) | 17 (8.6%) | 73 (36.9%) | 94 (47.5%) |
