# Supplementary material for: Poor Access for African Researchers to African Emergency Care Publications: A Cross-sectional Study
Source: West J Emerg Med. 2017 Sep 11;18(6):1018–24. doi: 10.5811/westjem.2017.8.34930 (PMC5654869; doi:10.5811/westjem.2017.8.34930)
Supplement: Supplementary file 2 [file wjem-18-1018-s002.docx]

Supplement B

| Supplement B. Breakdown of access to publications as compared between African and non-African first authors | | | |
| --- | --- | --- | --- |
| **Variable** | **All publications** | **African first author** | **Non-African first author** |
| All publications | 666 | 468 (70.3%) | 198 (29.7%) |
| Open access | 395 | 288 (61.5%) | 107 (54.0%) |
| Subscription-based | 271 | 180 (38.5%) | 91 (46.0%) |
| Archived/ self-archived | 106 | 65 (36.1%) | 41 (45.1%) |
| Not archived | 165 | 115 (63.9%) | 50 (54.9%) |
| Author provided | 60 | 40 (34.8%) | 20 (40.0%) |
| Author did not provide | 105 | 75 (65.2%) | 30 (60.0%) |
